# “I Can Also Serve as an Inspiration”: A Qualitative Study of the TB&Me Blogging Experience and Its Role in MDR-TB Treatment

**DOI:** 10.1371/journal.pone.0108591

**Published:** 2014-09-24

**Authors:** Shona Horter, Beverley Stringer, Sarah Venis, Philipp du Cros

**Affiliations:** Médecins Sans Frontières (MSF), London, United Kingdom; University of Stirling, United Kingdom

## Abstract

**Background:**

In 2011, Médecins Sans Frontières (MSF) established a blogging project, “TB&Me,” to enable patients with multidrug-resistant tuberculosis (MDR-TB) to share their experiences. By September 2012, 13 MDR-TB patients had blogged, either directly or with assistance, from the UK, Australia, Philippines, Swaziland, Central African Republic, Uganda, South Africa, India, and Armenia. Due to the lack of research on the potential for social media to support MDR-TB treatment and the innovative nature of the blog, we decided to conduct a qualitative study to examine patient and staff experiences. Our aim was to identify potential risks and benefits associated with blogging to enable us to determine whether social media had a role to play in supporting patients with MDR-TB.

**Methods and Findings:**

Participants were identified and selected purposively. TB&Me bloggers, project staff, MSF headquarters staff involved with TB and WHO European Region TB policy advisors were invited to participate in a semi-structured interview. Twenty interviews were conducted (five with bloggers). Data analysis drew upon principles of grounded theory, with constant comparison of data, cases and categories, and attention to deviant cases. We found that the TB&Me blog was associated with identified health benefits, with no reported instances of harm. There were three main findings: blogging was reported as useful for adherence to DR-TB treatment and supportive of the treatment-taking process by all bloggers and project staff; blogging provided support to patients (peer support, shared experience and reduction in isolation); and the blog was perceived as giving patients strength and voice.

**Conclusion:**

The TB&Me blog was seen to be associated with positive identified health and emotional benefits. Component 5 of the Stop TB Global Plan highlights the importance of empowering TB patients and communities. Blogging could be a useful tool to help achieve that ambition.

## Introduction

Multi-drug resistant tuberculosis (MDR-TB) is TB resistant to the two most powerful first-line anti-TB drugs: rifampicin and isoniazid. It is an increasing problem globally, with 630,000 estimated cases of MDR-TB among 12 million total cases of TB in 2011 [1]. Treatment for MDR-TB is lengthy, complex and can incur severe side-effects including deafness [2]. Adherence rates for treatment can thus be low [3]. In 2006, WHO launched the global *Stop TB Strategy*, as the internationally recommended approach to reducing the burden of TB. This strategy has six major components, of which component 5 is: *‘Empower people with TB, and communities through partnership’* [4]. There are a few examples of TB programmes that aim to give TB patients a voice in decision-making, in developing and implementing TB programmes or in taking more control of their care process [5]. However, general consensus about how TB patients can best be involved in treatment and care is lacking, and research into this area limited.

There are several examples of patients' involvement contributing to improved care provision, such as the development of cancer network partnership groups in the UK. These groups have been credited with enabling more effective and responsive cancer services through health-care professionals working with service users [6]. Patients have been said to use social media to change their interactions with providers, with technology supporting shared decision-making and facilitating communication between patients and providers, which enables patient-centred care and a shift towards treating the whole patient rather than just the disease [7].

There is now widespread and increasing access to the internet and use of social media, including in low and middle income settings. It is predicted that by 2015 over 3 billion individuals and businesses will have social networking accounts [8]. Mobile technology is increasing in reach with mobile phones frequently having internet connections, allowing those without computers to also access the web. Social media has been said to provide a useful tool for sharing health care information [8], [9], [10], with 61% of patients reportedly seeking support and medical information online [11].

Médecins Sans Frontières (MSF) established a blogging project in March 2011: TB&Me (http://blogs.msf.org/tb/), whereby MDR-TB patients around the world can document and share their experiences of living with the disease in order to raise awareness about MDR-TB. By September 2012, 13 MDR-TB patients had blogged on this website, either directly or with assistance, from the UK, Australia, Philippines, Swaziland, Central African Republic, Uganda, South Africa, India and Armenia. 10 of these 13 bloggers had received treatment and care for MDR-TB from MSF, with the remaining three being non-MSF patients.

The initial plan for the MSF blogging project was reviewed by the MSF Ethics Review Board as it was recognised that there could be potential harm as well as benefit for participants. There has been minimal research on this topic, particularly relating to TB, MDR-TB and patient perceptions around the potential benefits and risks of involvement with social media. Due to the emerging nature of social media and increased recognition of the supportive role it can play in health, the lack of existing research on the potential for this tool in supporting MDR-TB treatment and the innovative nature of the TB&Me blog, it was decided to conduct a qualitative study to examine patient and staff experiences of the blog. Our aim was to identify potential risks and benefits associated with blogging to enable us to determine whether social media had a role to play in supporting patients with MDR-TB.

### Background to the TB&Me project

When the blogging project was initiated in 2011, potential bloggers for the TB&Me site were identified by MSF project staff. In some cases they were patients who had already taken part in communication activities. After the project had started, certain TB patients not being treated by MSF contacted the project via the TB&Me website to express their interest in blogging on the site. Potential bloggers were given information about the blog, the blog's purpose and what blogging involved. Bloggers took part in the project voluntarily, with the project being clearly explained and informed consent being received prior to blogging commencement. MSF patients were informed that choosing not to take part in the blog or stopping at any point would not affect the services they receive in any way.

The blogging process was often assisted by project staff, for instance by collecting the blogger's response orally and transcribing it to the computer for those who were not computer literate or did not have access to a computer (subsequently referred to as “assisted bloggers”). Care was taken to ensure that the project staff member assisting the blogger was not directly responsible for the blogger's MDR-TB treatment. Bloggers who were not receiving TB treatment from MSF wrote blog entries themselves in English. Of the ten MSF bloggers, eight were assisted bloggers and six of the resultant blogs were translated into English. Blog entries were minimally edited to address only security concerns, defamatory comments or medically inaccurate information in addition to spelling and grammar. All non-minor changes were discussed with the patient and the patient's acceptance of the new version ensured before the blog was posted. Project staff would regularly provide feedback to assisted bloggers, including reading them any comments received on their blog entries.

The blog has provided a platform to reach a potentially wide audience. Over 50,000 people visited the TB&Me blog in the 8 months preceding September 2012, with 18% of visits occurring from mobile telephone access and most website visits occurring from the US (37%), UK (15%) and South Africa (10%). In September 2012 there had been 267 comments made on 94 posts on the blog alone, not including those on Facebook or Twitter, which are not as easily tracked.

## Methods

The study was conducted in September 2012. The protocol for the study is available at http://fieldresearch.msf.org/msf/handle/10144/241651. Participants were identified and selected purposively from three groups: all TB&Me bloggers; project staff who had been involved with the blogging project; and a stakeholder respondent group. Attempts were made to contact all 13 of the TB&Me bloggers, by either asking MSF project staff to request patients' permission to contact them with information about the study, or by sending information on the study to the patients directly in the case of non-MSF patients. Information about the study was sent to the project coordinators of all MSF projects where patients had been involved with blogging, requesting details of relevant staff to invite to interview. Project staff were invited to interview if they knew the patient blogger, potentially through supporting or treating them during their time blogging. Therefore insight into the project staff member's observed experience of the blog could be gained, as well as exploring whether they felt the patient had experienced any benefits or risks associated with blogging. Stakeholders were identified and invited to participate in an interview if their line of work linked to TB, policy and/or programming and if they had knowledge of the TB&Me blog. These interviews aimed to give insight into their views on potential strategic, programmatic and/or policy implications of the blog, as well as their views on the blog stories and access to the blog. This group were not directly involved in the blogging process and thus were not invited to discuss blogging experiences; rather, their technical opinions and views of the blog were sought.

Potential participants were invited to participate in a semi-structured interview. Interviews were audio recorded and all but one interview were conducted via Skype. Three interviews involved the use of an interpreter. Interpreters were briefed over Skype the day before the interview. Interviews lasted between 30 and 60 minutes with an average length of 45 minutes. Stakeholder interviews were generally of shorter duration than blogger or project staff interviews.

Interviews were conducted using a flexible participatory technique based on topic guides with prompts. Verbatim transcription was performed from interview audio recordings. Data analysis began with commencement of data collection, allowing an iterative process of data collection and analysis so that interview topic guides could be altered to further explore emerging themes [12]. Thematic analysis of transcripts was conducted to identify themes, patterns and concepts which emerged from the data to present the key elements of respondents' accounts [13]. Data analysis drew upon principles of the grounded theory method of analysis, with constant comparison of data, cases and categories to refine data and actively seek discrepancies between responses (deviant cases), thus ensuring findings were a true reflection of participant responses [14]. In addition, findings were triangulated from the blogger respondent group with those from the project staff group. During analysis, reflexivity about the influence of the researcher on shaping the data was ensured through awareness of potential researcher biases and analysing verbatim transcriptions word-for-word to avoid interpretation of meaning by the researcher. Coding and emergent themes were also checked by a second researcher to minimise potential bias. Attention was paid to fair dealing, with care taken to gain a range of perspectives from interviewing different groups of respondents.

### Ethics Statement

Formal ethics approval for the study was gained from the MSF Ethics Review Board. Informed consent forms were sent to participants prior to interviews with aid of an information sheet stating the purpose of the study and outlining the voluntary nature of participation. Informed, verbal consent was given by all participants prior to interview commencement and recording over Skype. Written consent was requested to be sent via post. Confidentiality was ensured through the use of pseudonyms and data storage protection procedures. Feedback mechanisms were used to ensure participants were aware of the findings of the study from which they could choose to opt out, this included sending a written summary of the findings and asking project staff to interpret or translate these for patients who spoke different languages or were illiterate. This consent procedure was approved by the MSF Ethics Review Board.

### Participants

Six of the 13 bloggers were un-contactable or untraceable: one had died, in another case the MSF project had closed and no contact details were available for the two patients; and one project (with three patients) required an additional local ethics approval process which could not be arranged within the study timeframe. All seven of the contactable patients were invited to participate: five agreed and were interviewed; the remaining two were interested in the study, but were not able to participate as they had finished treatment and now worked full-time. Of the five bloggers who participated: two were non-MSF patients who blogged directly, one was an MSF patient who blogged directly and two were MSF patients who were assisted bloggers.

Eight interviews were conducted with project staff (Table 1), both expatriate and nationals of the country, who had been closely involved with the bloggers. At least two staff members were interviewed from each interviewed blogger's location or project. Seven interviews were conducted with stakeholder respondents: two with WHO European Region TB specialists and five with various members of staff from MSF headquarters (Table 1). Further information on participants' geographic location and/or position of employment has not been provided to protect confidentiality. Thus, 20 interviews were conducted in total.

**Table 1:** Project staff and stakeholder participants.

Project Staff	
Role	No. Participants
Doctor	2
Nurse	2
Counsellor	3
Social worker	1

Stakeholders	
WHO/European Region TB	2
MSF/HQ General	2
MSF/HQ Public Health	3

HQ = headquarters.

## Results

Three key themes emerged from data analysis: participants found that blogging was useful for adherence to MDR-TB treatment, provided alternative support to patients and gave patients strength and voice. Triangulation of results showed that the predominant ideas and majority themes found in the blogger participant group were echoed in the project staff group. The responses of the stakeholder group supported some of the blogger and project staff responses, as well as providing insight into blog exposure and access.

### 1. Blogging and the treatment-taking process

All five blogger participants mentioned blogging as being supportive for adherence to MDR-TB treatment. Participants were not asked about this directly and thus it was a strong emergent theme, which was also reiterated by seven of the eight project staff respondents. The eighth project staff respondent did not mention adherence directly, but mentioned the blog as assisting with following infection prevention measures:

*‘The blog helped [patient]… to get motivated and use the mask’* Project Staff 01

Blogger participants mentioned the blog's comments facility as providing them with encouragement to continue with their treatment; as well as the feeling of having an audience following their blog whom they did not want to disappoint. This audience included other patients who they wanted to provide an example for:

*‘I said I will stop, I will not take the drugs anymore; but then I thought that I will disappoint all those who are following me on the blog at that point. And that's why I decided to continue with the treatment’* Blogger 05
*‘I was happy when I heard the words of encouragement from people, then I felt like to take the drugs as soon as possible’* Blogger 04

This was echoed by some project staff participant responses:

*‘That time his adherence was become very poor but… then we explained that see, all the world is following you and your blogs, and they are giving lots of good comments… after listening all these things then he came to me ‘yes, I can do these things, I will complete my treatment and I will show to this world that we can cure the TB’… and yes, then his adherence was good’* Project Staff 02
*‘On the blog people think that they are being watched and they are more likely to continue their treatment because of that… she understands that if she would write in the blog that she stopped the treatment it would influence other patients and it would have a negative effect on others’* Project Staff 07

Certain stakeholder responses also highlighted the potential for the blog to provide treatment support to patients, which was seen to be particularly significant for MDR-TB given the challenges associated with this treatment:

*‘It has helped them get across the darkest periods of their treatment… it might have encouraged them to not give up when they felt like giving up’* Stakeholder 05

Several participants saw the blog as providing a distraction from treatment, and some even felt blogging might help patients achieve positive health outcomes:

*‘And it [blog] helps her to deal with the treatment, to get cured from the treatment’* Project Staff 08

The struggles patients experience with MDR-TB treatment and the side effects are highlighted in several of the blogger stories, for example:

*I had hardly started the treatment when I began feeling terribly bad. After taking the drugs I was vomiting, losing my appetite, couldn't see or hear properly, had strange noises in my ears, felt a heaviness on my back, my heart was beating slowly and it was difficult for me to breathe.*
*After taking the drugs, these kinds of feelings started and lasted till evening. The doctors said I was feeling the “side-effects of the drugs” and I had to get used to them if I wanted to be cured.*
*After taking the drugs for two weeks and feeling like I was passing through hell, the only idea that occurred to me was to escape from the hospital. I was thinking that this kind of experience couldn't possibly be ‘treatment’. I began to think I would lose my mind or would die.* (Extract from blog story published on TB&Me)

Several reader comments have been made to blog stories encouraging patients to continue with their treatment, for example:

*One day at a time! Pain can make a slave out of a person, but you will be The winner – don't give up !!! You are not alone!!!* (reader comment on a blog story published on TB&Me)

### 2. Blogging providing alternative support to patients

Blogging was seen as providing support to bloggers and was also perceived as a source of peer support for other patients, by all blogger and project staff respondents and several stakeholder respondents. Participants mentioned the blog enabling patients to share their experiences with others, including other patients, project staff members and the general public:

*‘I'm kind of very happy about sharing experiences because people then they respond to the blog by writing some comments so you know it's like a conversation… you feel nice that somebody is listening to you and kind of feels what you have gone through’* Blogger 01
*‘She was very keen to have the opportunity to write her story and for other people to read it, that was happiness to her’* Project Staff 07

The blog was seen to provide bloggers with feelings of solidarity, reducing isolation through encouraging comments and through increased awareness of others experiencing MDR-TB:

‘[blogging] *makes you to realise that you are not alone in the struggle, that other people are also experiencing the same things that you are experiencing, and other people have already conquered the disease*’ Blogger 02
*‘when we see the blog, people are commenting on him, we printed out the email and gave it to him… he was so happy that tears flowed out because he said that ‘oh people are still loving me and still encouraging me to try more… even though I am alone or I am not with my family’… when he hears good comments from others it makes him strong’* Project Staff 06
*‘When you see that in different countries it's the same, everywhere, all patients can have the same difficulties it helps to understand that you are not the only one and as other people can overcome difficulties you also can do it’* Project Staff 08
*‘Solidarity. If I would be a TB or MDR-TB patient and I was reading this, I would feel encouraged… I would feel less alone that I have to take these 20 pills and my liver is a bit screwed up and… I'm really traumatised by the side effects, so I would see there are other people, and it's not in my country, it's in the region and globally, so I'm not alone… you contributed something to other patients… this kind of solidarity for the patient we should not underestimate’* Stakeholder 07

Participants felt that the blog was a source of peer support for other patients, providing them with hope and information about treatment and the disease:

*‘The main thing is to share my story because I understand the hardships of taking all those drugs and then the TB stigma and I think aside from being an inspiration for all those TB patients I can also serve as an inspiration for my co-bloggers at TB&Me because most of them are still under treatment’* Blogger 02
*‘The stories that you read on the blog are written by patients who passed already this way and they are successful, and this helps the patients who are just diagnosed with drug resistant TB to start the treatment’* Project Staff 07
*‘I can imagine that patients learn from each other's stories… I've seen that people who are blogging are looking at the stories from other people’* Stakeholder 04

This is also demonstrated through some of the comments on blog entries, where patients are reading other patients' stories:

*Hi, I am a patient of TB…. I am in the treatment of the MDR-TB… it's very hard to face the problems right now… therefore I started surfing web….* (reader comment on blog story published on TB&Me)

Several project staff and stakeholder interview respondents saw blogging as conducive towards the practitioner better understanding the patient, enabling enhanced patient-practitioner relationships and dialogue:

*‘Having the blog was really useful for us to get some kind of insight into what [patient] was thinking… [project staff members] realised some things about what she was thinking and feeling that they hadn't realised before’* Project Staff 04
*‘I think I gained trust and our relationship is stronger… this blog helped me personally as a [position] to know him better and… shows me how to deal with him with his problems’* Project Staff 03
*‘I would hope that there's a direct impact on field staff who are working with patients, you know, to have that patient connect and a better sense of what patients are going through’* Stakeholder 04

Beyond frontline practitioner relationships with patients, all seven stakeholder respondents mentioned the blog as providing a human face to MDR-TB, which enabled wider understanding of patients' experiences with the disease, and highlighted priority areas or key challenges for programmatic and policy focus:

*‘What is important in the blog is that… you don't see the patient as a patient, but as a personality and telling a story, and then it becomes very strong’* Stakeholder 03
*‘The connection with the patients and their stories and the understanding that side of it makes it more urgent and more real’* Stakeholder 04
*‘Social media is I think the best channel to get more MDR-TB patients involvement into defining the policy’* Stakeholder 06
*‘If patients are informing us through the blog about difficulties they're having with adherence, difficulties they're having with side effects, if it helps us to stop and think hey maybe we can do something about it to make it easier and not only for that patient but for other patients, then it stimulates ideas’* Stakeholder 04

There was one deviant case or discrepancy from the majority themes, where a blogger mentioned expectations of financial gain as a result of blogging. This concern was also raised by one of the project staff members working with this patient, who feared such expectations from the patient; but felt the informed consent process prior to blogging should have adequately achieved management of these expectations. This patient experienced memory loss as a side-effect of MDR-TB drugs, which might explain this response.

### 3. Blogging as a tool for patient expression and empowerment

Four of the five bloggers mentioned the blog as providing an avenue through which they could express their feelings and experiences, which was echoed by project staff responses. Several blogger participants saw this as preferable to communicating verbally, due to the journal-like nature of the blog providing ease of expression and less fear of judgement or audience reaction. The blog was said to offer the choice of whether or not to listen to or read blog audience reactions, whereas some participants felt when they discussed their experiences relating to MDR-TB verbally, the reactions they received could demonstrate a lack of understanding, empathy or support:

*‘Through blogging you can share thoughts without thinking of what others will think’* Blogger 02
*‘It helps to write what happened and what my experience was, what went on in terms of TB results, of the culture negatives and the positives’* Blogger 03
*‘I can imagine it being quite like, you know, like a diary to just get it all out there.’* Project Staff 04

The advantages of verbal expression, either through video or in person were mentioned by three blogger respondents, some project staff and several stakeholder respondents. These advantages included enhanced emotive response, ease of connection through eye contact and body language and allowing access to a different audience. These respondents felt that having the option of both blogging and verbal expression would be advantageous. Several barriers to verbal expression were mentioned: infection control; health status, for example patients unable to communicate verbally due to weakness or drug side-effects; language barriers; and fear of negative reaction or stigma.

*‘I love the fact that we do video blogging, this is much more effective in some cases at least, you know, you see people's facial emotions, you see their interaction, you see the happiness and despair in their faces, so it's probably much stronger’* Stakeholder 05
*‘TB is an airborne disease, and speaking face to face with an MDR-TB patient is not really safe. However, we can have face-to-face virtual discussion of speaking on the blog, sharing their experience’* Stakeholder 06

Several project staff and stakeholder respondents mentioned the blog as providing a voice for MDR-TB patients and a platform to speak out about their experience with the disease. The blog was identified as a tool through which patients could potentially be empowered, with sharing their experiences via this mechanism being seen as providing strength to the blogger:

*‘It will empower them and their health’* Blogger 05
*‘I mean obviously this kind of blog also… contributed to that, getting patients to have a voice’* Project Staff 04
*‘[the blog] Giving a stronger patient voice in a format that they don't have to directly say it to the doctor is very helpful’* Stakeholder 02
*‘The benefit that it directly gives to patients, that feeling of being empowered, that wanting to win their own you know struggle against the disease’* Stakeholder 05

It was also seen as a means by which patients could feel more positive, allowing them to reflect on the difficulties they had been through with MDR-TB. For patients who have completed treatment and are cured it gave them a sense of appreciation for what they have now. Some blogger and project staff respondents said it allowed recognition of their achievements:

*‘And they are like lessons in life, and you should never forget lessons, so it's kind of a going back to what you have gone through and in fact they make you even more strong. That you know, oh I have gone through this, what is this, what is this current situation, I can do this’* Blogger 01
*‘Now when I look back, whatever was written I have achieved it’* Blogger 05

Three of the five bloggers mentioned the blog as providing patients with a positive celebrity status, which was echoed by several project staff respondents:

*‘Now people around the world knows me because of my status’* Blogger 04
*‘She became a bit of a celebrity’* Project Staff 04

No patient or project staff respondents mentioned instances where patients involved with the blog experienced any negative outcomes or effects from the blog, nor perceived any harm subsequent to blogging.

## Discussion

We found that the TB&Me blog was associated with identified health benefits, with no reported instances of harm linked to blogging. The three main findings were:

- Blogging was found to be useful for adherence to MDR-TB treatment and supportive of the treatment-taking process.
- Blogging provided support to patients, including peer support, shared experience and enabling patients to overcome isolation.
- The blog was perceived as giving patients strength and voice.

This is presently the only study on the role of social media in supporting MDR-TB patients. In general, literature on the relation between blogging and the treatment-taking process or adherence is scarce. Of the existing literature on social media and health, studies have found a positive association between online social support participation and positive health outcomes [10], including blogging being associated with physiological and psychosocial well-being [15]. Several respondents in our study mentioned a perceived association between blogging and health outcomes, with support from blogging viewed as enabling improved treatment outcomes, converting to culture negative and being cured, as well as feeling more positive. Respondents in our study also found blogging to provide a distraction from the treatment taking process, which has been reiterated by other authors [16], who found that blogging could provide a distraction from a particular health condition. Our findings indicate the potential need for more adherence support measures within programmes, an area which warrants further investigation.

Respondents in our study associated the blog with providing patients with feelings of support, shared experience, not being alone and solidarity by connecting with other patients as well as through receiving encouragement and supportive words from blog followers. These findings have been reiterated by others, with studies finding health blogs to have the potential to serve as a means of acquiring social support and experiencing some of the health benefits associated with supportive communication and relationships [15]. Another study found the frequency of blogging predicted perceptions of social support and assurances of not being alone [16]. The internet is said to enable patients to share medical information, personal experiences and emotional sympathy with other patients who are suffering from a similar illness [10]. The fact that both assisted bloggers and those blogging directly both asserted these experiences with the blog implies that facilitating access to an online intervention such as the TB&Me blog for those who are not computer literate could potentially enable their engagement with an online community. Participants in our study also felt that the blog was a form of peer support, providing bloggers themselves and other patients with hope from reading about positive outcomes. Blogger participants felt the blog enabled them to help others by demonstrating the chance of recovery and providing tips and information on treatment and side-effects. Other studies have found that participants in online support groups felt they were helping others through sharing their stories and also became more optimistic about their own future by reading the stories of others who served as positive role models [17].

Our participants felt empowered by the blog as it aided recognition of their strength and achievements by enabling them to reflect on the journey they had been on and the struggles they had overcome. Other studies have also noted that social media can empower people [8], [10], [17] and that blogs enable people to easily measure their progress against their individual goals [18]. Several project staff and stakeholder respondents in our study felt that the blog provided patients with a voice and a platform for speaking out about their experience with MDR-TB. Others have argued that social media has the ability to turn communication into interactive dialogue [7], creating platforms to speak out [9], [19]. We found that blogging facilitated patients' expression, in part due to the journal-like nature of the blog, a finding echoed by other authors [15], [20]. There was less fear of judgement associated with blogging, the choice of anonymity and also the positive celebrity status participants mentioned highlights a potential role social media could play in stigma reduction. These findings are reiterated by others who have found the anonymous nature of online communities to allow patients to exchange personal concerns without fear of being judged [10]. In addition, being able to communicate without eye contact and the non-verbal responses of support providers can alleviate the embarrassment associated with disclosing undesirable information, particularly when related to stigmatised health conditions [15].

### Limitations

The methods inherent to qualitative study design limit the generalisability to concepts of our findings. However, participants were interviewed from a range of different countries and regions, with both MSF and non-MSF patients included, which enhances the validity and representativeness of the findings. There is a certain level of respondent bias in the selection of participants included in this study, in terms of which patients opted to blog. Only one form of social media was explored in this study; there is the need to look into the potential role of other social media tools.

It is unlikely that data saturation was achieved with five blogger interviews being conducted, however several steps were taken to ensure validity including triangulating blogger interview responses with those of project staff. As the same themes emerged from both respondent groups this strengthens validity of the findings. This study explored perceptions and experiences; it was not designed to gather evidence of whether the views expressed by staff and patients were reflected in real events. Patients' experiences can be very individual and varied; our findings that blogging was useful to these patients does not mean that blogging will be useful to all patients. Also, exploring the effect of the TB&Me blog on those reading the blog stories was outside of the scope of this study. The findings presented here reflect those of TB&Me bloggers, project staff and stakeholders working in the TB field who have prior knowledge of the TB&Me blog, rather than wider audiences or the general public. Exploring the influence of blogging on blogger readers could be a point for future research.

We have not distinguished between assisted bloggers and direct bloggers in the presentation of the findings, as the same themes emerged with both sets of participants. However, it should be noted that the additional support patients received related to blogging, particularly in the case of assisted bloggers, could have influenced the findings presented in this study.

## Conclusion

The findings suggest that the TB&Me blog was associated with identified health benefits, including reportedly supporting adherence to MDR-TB treatment; providing emotional and peer support; and providing strength and voice to patients. The steps taken to enhance the validity of our study findings mean that these findings are important, particularly if empowerment of patients is to be incorporated in health programmes as per component 5 of the Stop TB Global Plan [21]. Our study shows that blogging could be a useful tool to potentially contribute towards achieving that ambition. Our findings indicate the need for more adherence support measures within programmes, an area which warrants further investigation. At the very least, patient-centred care must involve the provision of accurate information to patients in easily digestible formats and the blogging medium could be ideal for this purpose.

There is the need for more research into the role social media could play within MDR-TB, as well as whether it could be likely beneficial in other areas of global health. However, this study highlights strongly the perceived benefits one social media tool brought to patients undergoing the lengthy, complex and toxic treatment regimen for MDR-TB.
